# *Pepino mosaic virus* genotype shift in North America and development of a loop-mediated isothermal amplification for rapid genotype identification

**DOI:** 10.1186/1743-422X-10-117

**Published:** 2013-04-12

**Authors:** Kai-Shu Ling, Rugang Li, Michael Bledsoe

**Affiliations:** 1 U.S. Department of Agriculture-Agricultural Research Service, U.S. Vegetable Laboratory, Charleston, SC 29414, USA; 2 Village Farms, Heathrow, FL 32746, USA

**Keywords:** *Pepino mosaic virus*, Genetic diversity, Genotype shift, Loop-mediated isothermal amplification

## Abstract

**Background:**

Pepino mosaic, once an emerging disease a decade ago, has become endemic on greenhouse tomatoes worldwide in recent years. Three distinct genotypes of *Pepino mosaic virus* (PepMV), including EU, US1 and CH2 have been recognized. Our earlier study conducted in 2006–2007 demonstrated a predominant EU genotype in Canada and United States. The objective of the present study was to monitor the dynamic of PepMV genetic composition and its current status in North America.

**Results:**

Through yearly monitoring efforts in 2009–2012, we detected a dramatic shift in the prevalent genotype of PepMV from the genotype EU to CH2 in North America since early 2010, with another shift from CH2 to US1 occurring in Mexico only two years later. Through genetic diversity analysis using the coat protein gene, such genotype shifting of PepMV in North America was linked to the positive identification of similar sequence variants in two different commercial tomato seed sources used for scion and rootstock, respectively. To allow for a quick identification, a reverse transcription loop-mediated isothermal amplification (RT-LAMP) system was developed and demonstrated to achieve a rapid identification for each of the three genotypes of PepMV, EU, US1 and CH2.

**Conclusion:**

Through systemic yearly monitoring and genetic diversity analysis, we identified a linkage between the field epidemic isolates and those from commercial tomato seed lots as the likely sources of initial PepMV inoculum that resulted in genetic shifting as observed on greenhouse tomatoes in North America. Application of the genotype-specific RT-LAMP system would allow growers to efficiently determine the genetic diversity on their crops.

## Background

Tomato (*Solanum lycopersicum* L.), an economically important vegetable, is widely grown around the world with leading tomato producing countries in China, the United States, Italy and Spain [1]. Although most of tomatoes are field-grown, protected production systems (greenhouse) have increased significantly in recent years, including in North America [2]. Pepino mosaic is one of several emerging viral diseases that seriously constrain the profitable production of tomatoes worldwide [3].

*Pepino mosaic virus* (PepMV) from the genus *Potexvirus* in the family *Alphaflexiviridae* was first reported to infect pepino (*S*. *muricatum* L.) in 1980 [4]. This virus was not considered an issue until 20 years later when it was first reported to infect greenhouse tomatoes in the Netherlands [5]. Since then, the disease has become widespread in Europe [6-14]. PepMV has also caused serious problems on greenhouse tomatoes in North America [15-18] and in South America [19-21]. Furthermore, this virus was detected in China [22], Syria [23], and South Africa [24]. Thus, PepMV has become one of the most economically important viruses infecting greenhouse tomatoes worldwide [3,25,26]. The typical disease symptoms on tomatoes include mosaic, yellow patches, necrotic lesions and uneven ripening of fruits resulting in marbling or flaming in appearance on mature fruits. It was estimated that the poor quality of PepMV-infected fruits could reduce tomato market value by up to 36% [27].

Three major genotypes of PepMV (namely EU, US1 and CH2), which share only 78-82% genomic nucleotide sequence identity, have been reported [9,10,16,21,25,26]. Even though a greater level of PepMV genetic diversity was initially recognized in the American continents [16,21], further population genetic analysis in Canada and the U.S. showed a predominant EU genotype, with only a small fraction of field isolates in US1 and CH2 genotypes [17]. However, several studies in Europe in recent years demonstrated that the prevalent PepMV genotype has shifted from a predominant EU [11,28] to CH2 [14,29-31]. Recent outbreaks of pepino disease in association with the CH2 genotype in broader geographic regions in the world, including the Middle East [23] and South Africa [24], raised concerns of possible seed transmission. Although PepMV on tomato is localized on seed coat (testa) and not in embryo, mechanical transmission from a contaminated seed could easily induce a new infection [32]. A low rate (0.026%) of PepMV seed transmission in tomato has already been observed [33]. These results on seed transmission demonstrated the importance of selecting and planting tomatoes from PepMV-tested negative seed lots. With intensive cultural practices, as required under a greenhouse tomato production system (i.e., grafting, de-leafing, inter-cropping and bumble bee pollination), even with a small number of PepMV-infected plants serving as sources of initial inocula could result in a serious disease epidemic. Thus, a timely detection and rapid genotype determination would be a prerequisite for deploying effective strategies in disease management.

Currently, several molecular-based methods have been developed to determine the genetic diversity of PepMV, including restriction fragment length polymorphism (RFLP) [34,35], reverse transcription- polymerase chain reaction (RT-PCR) and sequencing [14,17], real-time RT-PCR [36], and hybridization [30]. All such molecular methods required some specialized instruments or sophisticated laboratory conditions, making it difficult to apply for field analysis. With recent trends in genotype shifting in Europe, growers in North America are concerned about their crop’s health status and would request additional sequencing to determine the specific genotype [17]. Although this method is accurate, it is costly and time consuming. Thus, we were interested in developing a rather simple and quick reverse transcription loop-mediated isothermal amplification (RT-LAMP) for efficient identification of PepMV genotypes. In recent years, the RT-LAMP technology [37,38] has been successfully applied for plant virus and viroid detection [39-47]. In an effort to understand the current genetic diversity and evolution of PepMV in North America, we established a surveillance system with assistance from participating growers in Canada, Mexico and the U.S.A. on sample collection. We developed and used LAMP to monitor the presence and distribution of PepMV genetic diversity in North America.

## Results and discussion

### Development and validation of genotype-specific RT-PCR for PepMV

Through direct sequencing of RT-PCR amplified products and sequence analysis, specificity of the newly designed primers (Table 1) for genotype-specific amplification was evaluated on 50 field samples collected in North America in 2012 (Table 2). All 24 samples collected in Texas, USA were positive for CH2, while 10 of these samples were also of mixed infection with EU genotype but negative for US1. The 16 samples from Jocotitlan, Mexico were infected by the US1 genotype. Two among them were also of mixed infection with CH2 genotype. All 10 samples from British Columbia, Canada, were positive for CH2 by RT-PCR (Table 2).

**Table 1:** **Primer sequences for *Pepino mosaic virus *genotype-specific loop mediated isothermal amplification and RT-PCR**

**Genotype**	**Primer designation**	Sequence*	**Position on genome**	**GenBank accession #**
Primers for genotype-specific RT-LAMP				
CH2	CH2_F3	5^′^-CGATGAAGCTGAACAACATTTCC-3^′^	4297-4319	DQ000985
	CH2_FIP	5^′^-CTTAATGGGTTGATCTTGGTGGAAGCTGTGAGAAAGCTTCACAAAC-3^′^	4403-4381 + 4321-4343	
	CH2_BIP	5^′^-GGGTTAAGTTTTCCCCAGTTTGAAAATTCCTTCAGTGTTAATCTTGTG-3^′^	4404-4428 + 4498-4476	
	CH2_B3	5^′^-TCCAGCAATTCCGTGCACAACAA-3^′^	4523-4501	
	CH2_Loop F	5^′^-GGCCTCGCCTTGATGGA-3^′^	4360-4344	
	CH2_Loop B	5^′^-TGGAAAGATCAACTTTGATCAATT-3^′^	4429-4452	
EU	EU_F3	5^′^-ACCAAGAAGATACAAAATTTGC-3^′^	6090-6111	FJ940223
	EU_FIP	5^′^-TRAGACCATCAGCAGGCTGC TGCATTTGACTTCTTCGATG-3^′^	6173-6154 + 6112-6131	
	EU_BIP	5^′^-TCAGGCARCCAAATGAGAAAGAAACCTGTGGAGATCTTTTGC-3^′^	6174-6196 + 6256-6238	
	EU_B3	5^′^-TGACTTCTCCAAGTGTGG-3^′^	6284-6267	
	EU_Loop F	5^′^-TGGCAGGGTTGGTGACTC-3^′^	6149-6132	
	EU_Loop B	5^′^-CTAGCTGCTCACTCCGTAGCTAA-3^′^	6197-6219	
US1	US1_F3	5^′^-GCATTCATACCAAATGGGAG-3^′^	4255-4274	FJ940225
	US1_ FIP	5^′^-TGCGAACAGCCAAGAAATGT-ATAAATTGCATGAATACCTTACTCC-3^′^	4334-4315 + 4275-4299	
	US1_ BIP	5^′^-TTGCACAAACTCCACCAAGGACTTAACCCGTCAATGTGTT-3^′^	4337-4356 + 4415-4396	
	US1_ B3	5^′^-CCATTTCGAACAGGGGAA-3^′^	4433-4416	
	US1_ Loop F	5^′^-TGCTCAGCTTCATCA-3^′^	4413-4299	
	US1_ Loop B	5^′^-TGAAGCCATGAGACTT-3^′^	4357-4372	
Primers for genotype-specific RT-PCR				
CH2	PeppMVCH2CPF	5^′^- caggaaacagctatgacGTTTTCCTCAATTGTGAAAT-3^′^	5568-5587	DQ000985
	PepMVCH2CPR	5^′^- tgtaaaacgacggccagtTTTTTTTTTTATTTAGTAGATTTAGATAC-3^′^	6412-6394	
EU	PepMVEUCPF	5^′^ – caggaaacagctatgacGTTTTCCTAAATTTGAAAAT-3^′^	5572-5591	FJ940223
	PepMVEUCPR	5^′^ – tgtaaaacgacggccagtATTTCAAAGAAATAATTAGG-3^′^	6410-6391	
US1	PepMVUS1CPF	5^′^ - caggaaacagctatgacGTTTTCCTAGTGTTTGAAA-3^′^	5570-5588	FJ940225
	PepMVUS1CPR	5^′^ - tgtaaaacgacggccagtAAATTACAAAAGCAATTTATTG-3^′^	6415-6394	
Primers for full genome sequencing				
CH2	KL11-124:CH2-1F	5^′^- caggaaacagctatgacGAAAACAAAACATAACACATAATATC-3^′^	1-26	DQ000985
	KL11-131: CH2-1226R	5^′^- tgtaaaacgacggccagtTCATGCACCTCCAGTCATGT-3^′^	1226-1207	
	KL11-125: CH2-902F	5^′^- caggaaacagctatgacAAAAATAGCTTTGTGACCTTTCC-3^′^	902-924	
	KL11-132: CH2-1741R	5^′^- tgtaaaacgacggccagtGCTGGAAGTGTCAGATGCAA-3^′^	1741-1722	
	KL11-126: CH2-1395F	5^′^- caggaaacagctatgacCCAATTTAGTCAAACAAGGCGTA-3^′^	1395-1417	
	KL11-133: CH2- 2719R	5^′^- tgtaaaacgacggccagtAATTGGCACTTTGCACTTTTG-3^′^	2719-2699	
	KL11-127: CH2-2383F	5^′^- caggaaacagctatgacGATTCAACCTGGCTTTCCAA-3^′^	2383-2402	
	KL11-134: CH2- 3738R	5^′^- tgtaaaacgacggccagtGTTTGGGCGGTTCTGTTAAA-3^′^	3738-3719	
	KL11-128: CH2- 3379F	5^′^- caggaaacagctatgaCGACCTGGGAGATTTGTGCTG-3^′^	3379-3398	
	KL11-135: CH2- 4734R	5^′^- tgtaaaacgacggccagtGACAGGGGTCACCAAAAATG-3	4734-4715	
	KL11-129: CH2- 4381F	5^′^- caggaaacagctatgacTCCACCAAGATCAACCCATT-3^′^	4381-4400	
	KL11-136: CH2- 5748R	5^′^- tgtaaaacgacggccagtAAATCACTTAGGGAAGGAGCTG-3^′^	5748-5727	
	KL11-130: CH2- 5405F	5^′^- caggaaacagctatgacGCCGTAATATTCACCAGCATC-3^′^	5405-5425	
	KL11-137: CH2-6412	5^′^- tgtaaaacgacggccagtTTTTTTTTTTATTTAGTAGATTTAGATAC-3^′^	6412-6394	

*Primer adapters (M13 forward and reverse primer sequences, in lowercase) were added to specific viral sequences to facilitate direct sequencing of the amplified RT-PCR products.

**Table 2:** **Genotyping of *Pepino mosaic virus *isolates with reverse transcription loop-mediated isothermal amplification (RT-LAMP) and its validation with genotype-specific RT-PCR**

**Country of origin**	**Isolate name**	**ELISA**^**a**^	**EU**	**US1**	**CH2**
			**RT-PCR/RT-LAMP**	**RT-PCR/RT-LAMP**	**RT-PCR/RT-LAMP**
USA	VFTX12-01	(++++)	(−)/(+)^b^	−/−	+/+
	VFTX12-02	(++++)	(−)/(+)^b^	−/−	+/+
	VFTX12-03	(++++)	(−)/(+)^b^	−/−	+/+
	VFTX12-04	(++++)	−/−	−/−	+/+
	VFTX12-05	(++++)	+/+	−/−	+/+
	VFTX12-06	(++++)	+/+	−/−	+/+
	VFTX12-07	(++++)	+/+	−/−	+/+
	VFTX12-08	(++++)	+/+	−/−	+/+
	VFTX12-09	(+++)	+/+	−/−	+/+
	VFTX12-10	(++++)	+/+	−/−	+/+
	VFTX12-11	(++++)	−/−	−/−	+/+
	VFTX12-12	(++++)	−/−	−/−	+/+
	VFTX12-13	(+++)	+/+	−/−	+/+
	VFTX12-14	(+++)	+/+	−/−	+/+
	VFTX12-15	(+++)	+/+	−/−	+/+
	VFTX12-16	(+++)	−/−	−/−	+/+
	VFTX12-18	(+++)	−/−	−/−	+/+
	VFTX12-19	(+++)	−/−	−/−	+/+
	VFTX12-20	(+++)	−/−	−/−	+/+
	VFTX12-21	(+)	+/+	−/−	+/+
	VFTX12-22	(+++)	−/−	−/−	+/+
	VFTX12-23	(+)	−/−	−/−	+/+
	VFTX12-24	(+++)	−/−	−/−	+/+
	VFTX12-25	(+++)	−/−	−/−	+/+
Mexico	BNMX12-01	(+++)	−/−	+/+	−/−
	BNMX12-02	(+++)	−/−	+/+	−/−
	BNMX12-03	(+++)	−/−	+/+	−/−
	BNMX12-04	(+++)	−/−	+/+	−/−
	BNMX12-05	(+++)	−/−	+/+	−/−
	BNMX12-06	(+++)	−/−	+/+	−/−
	BNMX12-07	(+++)	−/−	+/+	−/−
	BNMX12-08	(+++)	−/−	+/+	−/−
	BNMX12-09	(+++)	−/−	+/+	−/−
	BNMX12-10	(+++)	−/−	+/+	−/−
	BNMX12-11	(+++)	−/−	+/+	−/−
	BNMX12-12	(+++)	−/−	+/+	−/−
	BNMX12-13	(+++)	−/−	+/+	+/+
	BNMX12-14	(+++)	−/−	+/+	−/−
	BNMX12-15	(++)	−/−	+/+	−/−
	BNMX12-16	(++)	−/−	+/+	+/+
Canada	VFBC12-01	(+++)	−/−	(−)/(+)^b^	+/+
	VFBC12-02	(++++)	−/−	(−)/(+)^b^	+/+
	VFBC12-03	(+++)	−/−	(−)/(+)^b^	+/+
	VFBC12-04	(−)	−/−	−/−	+/+
	VFBC12-05	(+++)	−/−	−/−	+/+
	VFBC12-06	(++)	−/−	−/−	+/+
	VFBC12-07	(+)	−/−	−/−	+/+
	VFBC12-08	(++++)	−/−	−/−	+/+
	VFBC12-09	(+)	−/−	−/−	+/+
	VFBC12-10	(+++)	−/−	−/−	(+)/+^c^
Positive		(+++)	+/+	+/+	+/+
Negative		(−)	−/−	−/−	−/−

^a^ ELISA ratings: (−) <0.100; (+): 0.101-0.500; (++): 0.501-1.000; (+++): 1.001-2.000; (++++) >2.001.

^b^ A discrepancy was observed in these isolates with a negative (−) RT-PCR, but a positive (+) RT-LAMP. A confirmation test through sequencing of cloned RT-LAMP products was performed to determine the authenticity of their viral origin.

^c^ The result for a positive (+) RT-PCR was not consistent from one experiment to another while RT-LAMP was consistently positive.

### The sensitivity and reliability of RT-LAMP in genotype determination of PepMV isolates

**Figure 1:**
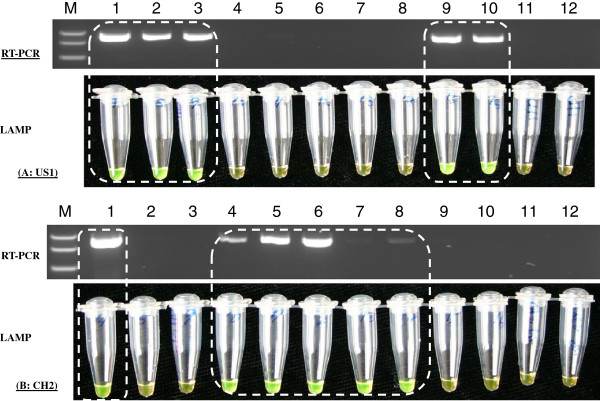
**Comparative analysis of a genotype-specific detection of *Pepino mosaic virus *isolates using RT-LAMP and RT-PCR.** The upper panel (**A**) was for genotype US1 isolates. The lower panel (**B**) was for genotype CH2 isolates. Lane M: PCR marker; Lane 1 was a positive control in a mixed infection with two genotypes of PepMV (US1 and CH2); Lanes 2–10 were 9 field samples infected with PepMV; Lane 11 was a health tomato (negative) control; Lane 12 was a non-template (blank) control. PepMV isolates in Lanes 2, 3, 9 and 10 were typed as US1. The isolates in Lanes 4–8 were shown to be CH2 type. Note, the faint products in lanes 7–8 by RT-PCR, were clearly visible by RT-LAMP.

The specificity of the developed RT-LAMP for each genotype (including EU, US1 and CH2) was validated with a genotype-specific RT-PCR (Figure 1 and Table 2). A greater sensitivity was obtained for RT-LAMP than that of its respective RT-PCR, as demonstrated with two CH2 isolates (Figure 1). Consequently, these genotype-specific RT-LAMPs were used to evaluate field samples for their genotype composition, confirming a single or a mixed infection of two or three genotypes (Table 2). Although test results using genotype-specific RT-LAMP and RT-PCR on 44 of 50 field collected samples were in full agreement with one another, there were some minor discrepancies in six other samples (Table 2). RT-LAMP was able to detect the presence of genotypes CH2 and EU sequences in three Texas samples (isolates, VFTX12-01 to VFTX12-03); whilst only CH2 was detectable by RT-PCR in the same samples. Similarly, both CH2 and US1 sequences could be identified by RT-LAMP in three Canadian samples (isolates: VFBC12-01 to VFBC12-03); RT-PCR, however, only detected the presence of genotype CH2. To resolve such discrepancies, sequences from cloned RT-LAMP products were obtained and validated. Thus, RT-LAMP demonstrated a greater sensitivity than RT-PCR in detecting such low concentration of the virus.

### The prevalent PepMV genotype shifted in North America

PepMV isolates collected from selected greenhouses in Canada, Mexico and the U.S. were genotyped over seven consecutive years (2006–2012). Prior reported genotyping data from an imported seed sample from Chile [21] and the survey and genotyping results in 2006–2007 [17] served as base lines to evaluate the dynamic of genotype shift in North America. Prior to 2009, prevalent isolates of PepMV in Canada and the U.S. were predominantly in the EU genotype. Surprisingly, a shift in the prevalent genotype was observed in early 2010 when all 10 samples (four from Canada, three from Mexico and three from the U.S.) were shown to be CH2. Six additional samples (five from Mexico and one from the U.S.) collected in 2011 also belonged to genotype CH2 (Table 3). To better assess the genetic diversity of PepMV, a larger number of samples were collected and analyzed in 2012; 10, 16, and 32 samples were collected from Canada, Mexico, and the U.S. greenhouses, respectively. Greater variations in the genetic diversity were observed in the three countries (Table 3). CH2 sequences were detected in all 10 samples from Canada. Among them, two were in a mixed infection of EU. In the U.S., 31 of 32 samples analyzed contained the CH2 genotype sequence, with 17 in a single infection and 14 a mixed infection of CH2 and EU. Only 1 of 32 samples was singly infected by EU (Table 3). In contrast, 16 of 22 samples collected from Mexico in 2012 were genotyped as US1, with 14 of them containing a single infection and the other two a mixed infection of US1 and CH2 (Table 2). These results indicated a genotype shift from CH2 to US1 in Mexico. All sequence data were deposited in GenBank [JX866611 - JX866663].

**Table 3:** **Genetic diversity and dynamic of *Pepino mosaic virus *genotypes in North America from 2006 to 2012**

**Year**	**Canada**^**a**^	**Mexico**	**U.S.A.**	**Reference**
2006	EU (4/4)	nt^b^	EU (7/7)	Ling et al., 2008 [17]
2007	nt	nt	EU + CH2 (1/1)	
2009	nt	0/10	EU (4/4)	This study
2010	CH2 (4/4)	CH2 (3/3)	CH2 (3/3)	
2011	nt	CH2 (5/5)	CH2 (1/1)	
2012	CH2 (7/10), US1 + CH2 (3/10)	US1 (14/22), CH2 (6/22), US1 + CH2 (2/22)	CH2 (17/32), CH2 + EU (14/32), EU (1/32)	

^a^ Greenhouse location: Canada (Delta, British Columbia), Mexico (Jocotitlan, Mexico) and USA (Marfa, Texas). The number inside each parenthesis represents the number of PepMV isolates in that genotype/total number of samples submitted by growers for PepMV identification and genotype examination.

^b^ nt: not tested.

### The sequence identity of field collected PepMV isolates to those from commercial tomato seed lots

Analysis of the PepMV genetic diversity using the complete coat protein or partial RNA-dependant RNA polymerase (RdRP) genes showed the presence of quasispecies nature of PepMV in field populations throughout North America. Percentages of each sequence variants based on the coat protein gene were determined upon careful analysis to a multiple sequence alignment using 9 isolates from Canada and 27 isolates from the U.S. Despite such genetic diversity, the coat protein gene sequences among variants within the same genotype were very similar. There was only less than 1.0% sequence diversity among isolates in the PepMV CH2 genotype. A different gene (partial RdRP) was used to validate the genetic diversity of PepMV CH2 genotype as observed in 10 isolates from the U.S. [GenBank: JX866669 - JX866678]. Interestingly, the predominant CH2 variant (JX866670) shared an identical RdRP sequence to that of PepMV in a commercial tomato seed sample [‘F1Seed1’, VFTX12-32, GenBank: JX866669]. Among the 22 sequence variants from Texas, two major variant types were identified. Although the majority of CH2 variants from Texas (18 of 22) contained a typical coat protein gene (714 nt), 4 other sequence variants had an unique deletion of 12 nt (−GCTTCTAACCCA-) in the 5^′^ terminal portion of the coat protein gene between nt 19–30 resulting in a deletion of 4 aa (−ASNP-). Surprisingly, the same two major sequence variants were also identified in the commercial F1 hybrid tomato seed sample ‘F1Seed1’, including a normal coat protein gene [GenBank: JX866657] and a coat protein gene with 12 nt deletion [GenBank: KC579401]. A similar genetic diversity was also observed within the coat protein of 15 isolates in a different genotype (US1) from Mexico in 2012. Interestingly, a US1 isolate identified in another commercial tomato rootstock seed ‘RSSeed2’ [GenBank: JX8666635] was nearly identical to the 15 isolates from greenhouse tomatoes (GenBank: JX8666620-JX8666634). Such genetic diversity analysis strengthened the link between those of field epidemic isolates and those from commercial tomato seed lots, stressing the importance of planting PepMV-tested negative seed lots to reduce the chance of introducing contaminated planting materials into a production greenhouse.

### Phylogenetic relationship and genome sequencing to the emerging genotypes

**Figure 2:**
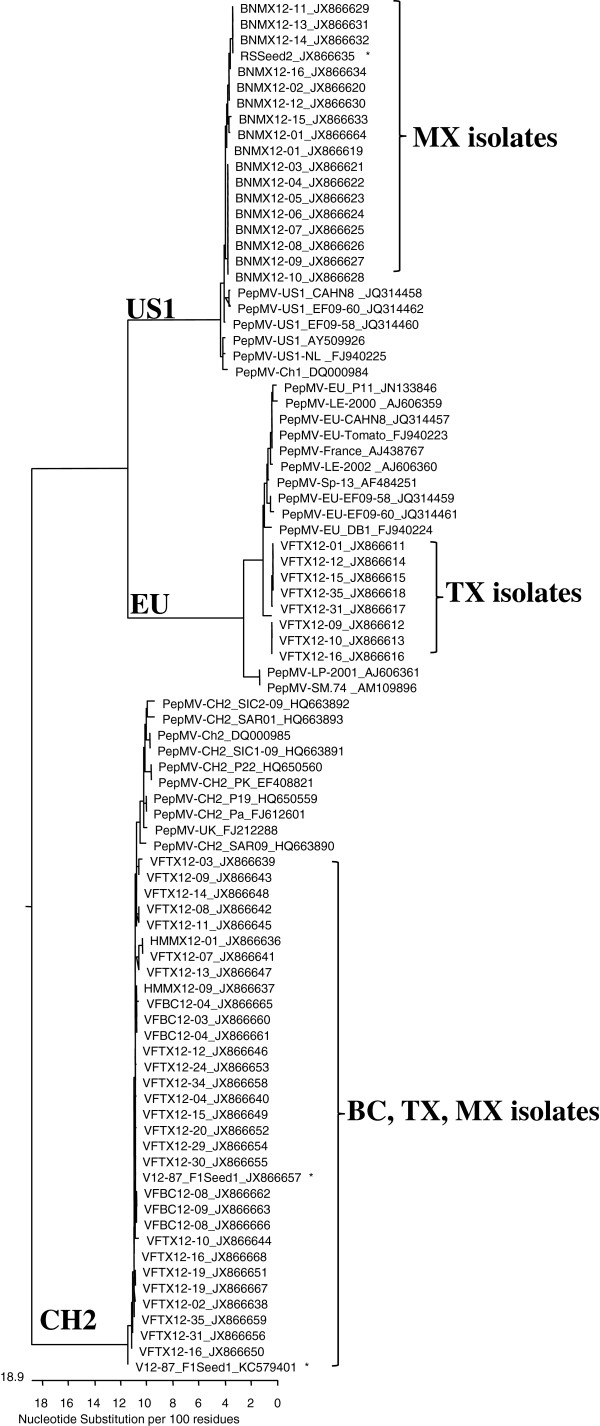
**Phylogenetic relationship of *Pepino mosaic virus *isolates analyzed using the coat protein gene sequences.** The GenBank accession numbers for each virus isolate used were included in each entry. The 16 PepMV isolates collected from Mexico (MX) named as BNMX12- [GenBank: JX866619 – JX866634] clustered with the US1 lineage along with the isolate recovered from a commercial rootstock seed lot designated as ‘RSSeed2’, [GenBank: JX866635]. The two additional Mexican (MX) isolates in the CH2 lineage designated as HMMX12- [GenBank: JX866636 – JX866637] were collected from a different greenhouse in Mexico. The four Canadian (BC) isolates in the CH2 genotype are designated as VFBC12- [GenBank: JX866660 – JX866663]. The U.S. (TX) isolates shown as VFTX12-, including eight isolates in the EU genotype [GenBank: JX866611 – JX866618] and 22 representative isolates in the CH2 genotype [GenBank: JX866638 – JX866659], clustered along with two CH2 sequence variants recovered from a commercial tomato seed ‘F1Seed1’ [GenBank: JX866657 in a normal CP gene and KC579401 in a 12-nt deletion mutant]. For better identification, the three sequence variants obtained from the two seed samples were labeled with asterisks.

A phylogenetic tree was constructed by incorporating full coat protein genes from 53 PepMV isolates sequenced in the present study along with previously published PepMV sequences in GeneBank. As shown in Figure 2, all newly identified PepMV isolates could be assigned into three distinct phylogenetic lineages (or genotypes). The 16 Mexican isolates [with designations beginning with BNMX12-, GenBank: JX866619-JX866634] were grouped with the genotype US1. Eight U.S. isolates [with designations beginning with VFTX12-, GenBank: JX866611-JX866618] belonged to the genotype EU whereas another 28 isolates, including 22 from the U.S. [with designations beginning with VFTX12-, GenBank: JX866638-JX866659], four from Canada [with designations beginning with VFBC12-, GenBank: JX866660-JX866663), and two from a second location in Mexico [with designations beginning with HMMX12, GenBank: JX866636-JX866637), were found to belong to the CH2 genotype. All 10 Canadian isolates were assigned to the CH2 genotype but the U.S. isolates were separated in the CH2 and EU lineages. These CH2 variants from seed ‘F1Seed1’ [GenBank: JX866657 and KC579401] were clustered with other CH2 isolates from North American greenhouses. Although PepMV isolates from Mexico analyzed in the 2010 and 2011 growing seasons were genotyped as CH2 (Table 3), additional 16 isolates collected in 2012 from the same greenhouse belonged to genotype US1.

To gain better insights into these prevalent genotypes (CH2 and US1), extra efforts were taken to fully sequence the virus genomes in several selected isolates for comparison. These isolates included one US1 isolate from Mexico [BNMX12-01, GenBank: JX866664], two CH2 isolates from the U.S. [VFTX12-16, GenBank: JX866668; VFTX12-19, GenBank: JX866667] and two other CH2 isolates from Canada [VFBC12-04, GenBank: JX866665; VFBC12-08, GenBank: JX866666]. Sequence analysis demonstrated that the four CH2 isolates had 99.8% nucleotide sequence identity to one another but only 98.2% identity to the original Ch2 isolate [GenBank: DQ000985]. The two CH2 isolates from the U.S. (VFTX12-16 and VFTX12-19) with a 12-nt deletion in the coat protein gene were confirmed upon full genome sequencing.

Our previous surveys and genotyping efforts conducted in the 2006–2007 growing season showed a predominant EU genotype in Canada and the U.S. [17]. The CH2 genotype in the present study was consistently detected in grower’s samples from Canada and the U.S. since early 2010. Although the first natural PepMV infection was observed in 2010 in Mexico [18], the CH2 genotype was prevalent there for only two years until 2011 (Table 3), before being displaced by the genotype US1 in 2012 (Figure 2).

Although seed transmission of PepMV in tomato may occur sporadically due to the virus being localized on the seed coat (testa) and not in the embryo [32], tomato seedling infection can occur either through seedling germination [33] or by mechanical inoculation [32]. However, how these PepMV CH2 isolates were initially introduced into European greenhouse tomatoes was not clear [14,29-31]. It is possible that these CH2 isolates could be introduced in the same manner as those described here, because many European greenhouse tomato growers use similar sources of commercial tomato seeds as American growers. Moreover, sequences from these European CH2 isolates are very similar to those from North America as indicated in the phylogenetic analysis (Figure 2). The Chilean isolates (Ch1 = US1 genotype, Ch2 = CH2 genotype) were identified in a commercial seed lot imported from Chile in 2004 [27]. A CH2 isolate was also detected in 2007 in Texas [17]. In just a few years, the CH2 genotype replaced the EU genotype and emerged as a predominant strain in causing pepino mosaic disease epidemics in many greenhouse tomato facilities around the world. Gomez and colleagues [30] demonstrated a higher genetic fitness for a CH2 isolate in a mixed infection of an EU isolate. The data from the current study appeared to support such notion in that the titer of EU in the three Texan samples (VFTX12-01 to VFTX12-03) mixed infected with CH2 was so low that it was not detectable by RT-PCR. Only after the use of more sensitive RT-LAMP was the presence of EU sequence confirmed (Table 2). Similarly, in three Canadian samples (VFBC12-01 to VFBC12-03) containing a mixed infection of CH2 and US1, the presence of US1 sequence was detected only by the use of RT-LAMP (Table 2). Such genotype displacement could also be enhanced by several environmental and ecological factors associated with intense greenhouse tomato production system. In addition, the human factor in performing intensive hands-on operations can play an important role in PepMV transmission once introduced in a greenhouse facility. Despite the increasing demand and requirement in seed health test for PepMV, the current standard seed health test as recommended by International Seed Health Initiative (ISHI) with ELISA test may not be sensitive enough to detect a low level of seed contamination in commercial seed lots. In two seed samples that were initially negative for PepMV in ELISA, the virus was revealed by immunocapture real-time RT-PCR [48]. Although the Ct values were close to the upper end of the “positive” cutoff (Ct = 35), with Ct = 33.67 for ‘F1Seed1’ and Ct = 32.24 for ‘RSSeed2’while the PepMV-positive control seed had a Ct = 25.53. Subsequently, positive identification was confirmed using the genotype-specific RT-PCR system through direct sequencing and sequence analysis of the amplified RT-PCR product.

The three major genotypes of PepMV (EU, US1 and CH2) have been recognized in North America [16,17,21]. However, we could not confirm the presence of US2 genotype either through Sanger sequencing or by small RNA deep sequencing [49]. The original US2 genotype sequence [16] was probably either a natural recombinant or an artifact between the two recognized genotypes (US1 and EU), and thus was not considered further as one of the representative genotypes for PepMV. We also did not detect the so-called LP genotype [12] in our surveys of PepMV on commercial greenhouse tomatoes in North America.

The LAMP technology is highly specific for the target sequence and has been developed as a powerful tool for viral disease diagnosis [39-47]. In the present study, we were interested in developing genotype –specific RT-LAMP that would permit an effective and efficient genotyping without lengthy sequencing. Initial genotype analysis was performed using sequencing of RT-PCR products containing a small portion of the viral genome [17]. This sequencing method is accurate, but is laborious and time consuming. To allow for an efficient assessment of many field-collected samples, it was necessary to develop and apply a simple technology. The genotype-specific RT-LAMP developed in the current study was effective for PepMV genotyping and its specificity was validated using RT-PCR and sequencing. Although the amplified RT-LAMP products can be detected in a number of ways, in the current study, we chose a direct staining with SYBR Green in an enclosed tube to minimize potential cross-contamination. Thus, the genotype-specific RT-LAMP technology could be useful in field setting to analyze PepMV isolates for their genetic composition.

The highly contagious nature of PepMV makes it extremely difficult to control. Cross-protection has been shown to be an effective measure only if it is used against a virulent isolate belonging to the same genotype [50]. Cross-protection is not effective against a PepMV isolate in a different genotype and could potentially result in even more serious disease in a mixed infection [15]. Therefore, understanding the genetic diversity is critical in deciding which mild strain of the virus should be used to achieve a successful cross protection.

## Conclusions

The population genetic analysis in the present study showed a shift in the prevalent genotype of PepMV from the EU to the CH2 genotype since 2010 in North America. This result is in agreement with the trend observed in several European countries [14,29-31]. In the present study, we revealed another genotype shifting event from CH2 to US1 in Mexico only two years after the first identification of PepMV infection there [18]. Such genotype shift was likely not induced through a natural virus mutation in such a short period of time but rather from an unintentional introduction of a more aggressive genotype of PepMV through the use of contaminated tomato seed lots. This hypothesis was supported by the high sequence identity between the PepMV isolates originated from greenhouse tomatoes and those from commercial tomato seed sources (scion and rootstock). Taken together, our genetic diversity characterization and phylogenetic analysis supported the notion that contaminated tomato seed lots were the sources of initial inocula for disease epidemic, which resulted in a dramatic genotype shifting in North America from one year to another. The application of genotype-specific RT-LAMP can enhance our capacity to monitor field genotype composition and to apply appropriate strategies to manage pepino mosaic disease.

## Materials and methods

### Survey, culture collection and detection of PepMV

In collaboration with three major greenhouse tomato growers in North America, we initiated a surveillance system to monitor the occurrence of PepMV in Canada, Mexico and the United States and to characterize the genetic diversity of field populations of PepMV. After collection, samples were shipped to our lab at the U.S. Vegetable Laboratory (Charleston, SC, USA) using appropriate plant pathogen diagnostic permits. Upon receipt, samples were inspected for symptom expression and tested for the presence of PepMV by enzyme-linked immunosorbent assay (ELISA) (Agdia, USA), AgriStrip (BioReba, Switzerland), or immunocapture real-time RT-PCR [48]. Occasionally, virus infectivity was assayed by mechanical inoculation of tomato or *Nicotiana benthamiana* plants [32]. The collected tissue samples were at different stages of plant development with symptoms ranging from foliar mosaic to marbling on fruits. Thus, the virus isolates reported in this study were representative of a broad spectrum of PepMV populations in these greenhouses. To characterize genetic diversity of PepMV and to monitor a dynamic of genotype shift over the years, tomato samples collected and tested positive for PepMV by ELISA from the same representative greenhouse in each of the three countries in North America were used for molecular characterization. In the present study, fourteen samples originated from Canada, four collected in 2010 and ten in 2012. Twenty four samples were collected from Mexico, three in 2010, five in 2011, and sixteen in 2012. Forty samples were from the United States, including four in 2009, three in 2010, one in 2011, and thirty two in 2012. In addition, two commercial tomato seed samples (designated as ‘F1Seed1’ for a F1 hybrid and ‘RSSeed2’ for a rootstock) were a gift from an anonymous donor. According to the International Seed Health Initiative standard method for PepMV [51], seed health tests were performed with 3,000 seeds divided into 12 subsamples of 250 seeds each using ELISA (Agdia, USA) or immunocapture real-time RT-PCR [48].

### Development of genotype specific RT-PCR

Initial genotype analysis for PepMV was based on sequencing the amplified RT-PCR products using two sets of primers targeting two genomic regions to allow for accurate genotyping [17]. The first generic primer pair KL05-13 (5^′^ GTC CTC ACC AAT AAA TTT AG 3^′^) and KL05-14 (5^′^ AGG AAA ACT TAA CCC GTT C 3^′^) generated a relatively short genomic sequence (202 bp) targeting the TGB2-3 region which was useful for general PepMV genotyping among EU, US1 and CH2. The second primer set KL04-52 (5^′^ GTG CTT ACA GTT CTG ACA TC 3^′^) and KL05-20 (5^′^ CTA GAA TTG GCA CTT TGC AC 3^′^) targeting the CH2 genome in the RNA dependant RNA polymerase (RdRp) region were used to confirm the identification of a CH2 isolate [17]. Therefore, this process was cumbersome and time consuming. To allow for more efficient genotyping, three genotype-specific primer sets were designed based on the conserved sequence region in each genotype in this study (Table 1). Using these primer sets, RT-PCR amplified products contained sequences of the coat protein gene and a portion of 3^′^ untranslated region for each specific genotype. To facilitate direct sequencing of the RT-PCR products, primers were designed to contain the M13 forward or reverse primer sequences. RT-PCR was conducted using a template viral RNA from immunocapture [48], or with a total plant RNA prepared using an RNeasy plant kit (Qiagen, USA) or a TRIzol reagent (Invitrogen, USA). RT-PCR was conducted using a Superscript III one-Step RT-PCR kit with RT/Platinum Taq Hifi enzyme mix (Invitrogen, USA). The cycling parameters included a reverse transcription for 30 min at 50°C and a denaturation for 2 min at 94°C, followed by 40 cycles of denaturation at 94°C for 30 sec, annealing at 55°C for 30 sec and extension at 72°C for 1 min, with a final extension cycle of 10 min at 72°C. An aliquot of RT-PCR preparation (10 μl) from each reaction was applied onto a 1.5% agarose gel for electrophoresis and the RT-PCR products were visualized under a UV transilluminator after staining with 0.01% SYBR Safe (Invitrogen, USA). Gel images were taken using a ChemiDoc digital imaging system (Bio-Rad, USA). To confirm the genotype specificity, nucleotide sequencing was conducted. Upon purification using a QIAquick PCR Purification kit (Qiagen, USA), RT-PCR products were used for direct sequencing or cloned into a pCR4-TOPO vector (Invitrogen, USA). Sanger sequencing was performed by Functional Genomics (Madison, WI, USA). Sequence assembly and alignment analysis were performed with DNASTAR Lasergene 10 (Madison, WI, USA). In addition, complete genomic sequences from selected isolates representing CH2 or US1 were determined using overlapping RT-PCR products generated with genotype specific primers for CH2 (Table 1) or for US1 [49].

### Reverse transcription loop-mediated isothermal amplification (RT-LAMP) for genotype-specific detection

Through multiple alignments of complete genomic PepMV sequences, a conserved region (~99%) among isolates within each particular genotype (EU, US1, or CH2) was identified. Using consensus sequences from the identified conserved region (about 200 nt), a set of primers suitable for RT-LAMP was designed using PrimerExplorer V4 software [52] and synthesized by Sigma Genosys (USA) (Table 1). The RT-LAMP assay was conducted using a RNA amplification kit from Eiken Chemical (Japan) following the manufacturer’s instructions. Each 25 μl reaction consisted of 12.5 μl of 2× reaction buffer [containing 40 mM Tris–HCl (pH 8.8), 20 mM KCl, 16 mM MgSO_4_, 20 mM (NH_4_)_2_SO_4_, 0.2% Tween 20, 1.6M Betaine, and 2.8 mM each of dNTPs], 2 μl of 20 μM forward inner primer (FIP), 2μl of 20 μM back inner primer (BIP), 1 μl of 20 μM of Loop-F primer, 1μl of 20 μM of Loop-B primer, 0.25 μl of 20 μM of F3 primer, 0.25 μl of 20 μM of B3 primer, 1.0 μl of enzyme mix (reverse transcriptase and DNA polymerase) and 2.0 μl of RNA preparation. The mixture was incubated at 65°C for 60 min, followed by a denaturation for 2 min at 95°C to terminate the reaction. Products were checked by electrophoresis on a 2% agarose gel containing 1:10,000 diluted SYBR gel stain or directly visualized by addition of 1.0 μl of 1:10 diluted SYBR Green I (Invitrogen, USA). In some cases, to validate the sequence specificity, RT-LAMP products were cloned with a TOPO TA cloning kit (Invitrogen, USA) after tailing with dATP in 1x GoTag buffer (Promega, USA) at 72C for 15 minutes. Upon screening, colonies with inserts were selected, sequenced and analyzed using BLASTn in the National Center for Biotechnology Information (NCBI) database.

### Phylogenetic analysis

To assess genetic composition of PepMV populations in samples collected from North America, the coat protein gene region were aligned and analyzed with CLUSTAL-W [53]. A phylogenetic relationship for these PepMV isolates was assessed using the neighbor-joining method [54] in DNASTAR Lasergene 10 in 1,000 bootstrap iterations to generate a robust phylogenetic tree.

## Abbreviations

PepMV: *Pepino mosaic virus*; ELISA: Enzyme-linked immunosorbent assay; RT-PCR: Reverse transcription polymerase chain reaction; RT-LAMP: Reverse transcription loop-mediated isothermal amplification; ISHI: International Seed Health Initiative; RdRP: RNA-dependant RNA polymerase; NCBI: National Center for Biotechnology Information.

## Competing interests

The authors declare that they have no competing interests.

## Authors’ contribution

KSL conceived the study, designed methods and experiments; performed experiments and sequence analysis; analyzed data and interpretation, drafted and revised the manuscript. RL designed primers for RT-LAMP; performed RT-LAMP; conducted sequence analysis; evaluated data and revised the manuscript. MB coordinated activities in greenhouse survey, sample collection, and revised manuscript. All the authors have read and approved the final manuscript.
